# Advances in Neurodegenerative Disease Therapy: Stem Cell Clinical Trials and Promise of Engineered Exosomes

**DOI:** 10.1111/cns.70577

**Published:** 2025-09-04

**Authors:** Sevim Isik, Sajeda Osman, Bercem Yeman‐Kiyak, Suhair Rami Mohammed Shamshir, Nesrin Majdi Edwan Sanchez

**Affiliations:** ^1^ Department of Molecular Biology and Genetics Faculty of Engineering and Natural Sciences, Uskudar University Uskudar Istanbul Turkey; ^2^ Stem Cell Research and Application Center (USKOKMER), Uskudar University Uskudar Istanbul Turkey; ^3^ NPCELLAB Cellular Therapy, Stem Cell and Advanced Therapy Medicinal Products GMP Laboratory Uskudar University Uskudar Istanbul Turkey; ^4^ Department of Molecular Biology, Institute of Science Uskudar University Uskudar Istanbul Turkey

**Keywords:** alzheimer's disease, amyotrophic lateral sclerosis, cellular therapy, clinical trials, exosomes, huntington's disease, parkinson's disease, stem cells

## Abstract

**Aim:**

This review provides a systematic evaluation of 94 stem cell clinical trials to treat neurodegenerative diseases, including Alzheimer's disease, Parkinson's disease, amyotrophic lateral sclerosis, and Huntington's disease.

**Methods:**

Data were collected from using relevant search terms, focusing exclusively on stem cell therapy. Of the 8000+ participants in these trials, nearly 70% were enrolled in AD‐related studies. Only three Phase 3 studies were conducted, and most trials were in the early phases (Phases 1 and 2). Mesenchymal stem cells, neural stem cells, induced pluripotent stem cells, and embryonic stem cells are used the most to treat neurodegenerative diseases. This review also explores the emerging fields of preclinical and clinical investigations of stem cell‐derived exosome‐based therapies for neurodegenerative diseases.

**Results:**

Exosomes can cross the blood–brain barrier to deliver therapeutic molecules directly to the brain, offering a less invasive alternative to stem cell transplantation. Mesenchymal stem cell‐derived exosomes, in particular, have demonstrated significant potential in preclinical models by reducing neuroinflammation, oxidative stress, and promoting neuronal regeneration. Additionally, recent advances in exosome engineering, including surface modifications, therapeutic agent loading, and transgenic modifications, have improved targeting, stability, blood–brain barrier delivery, and neural cell interactions, enabling targeted and effective treatment. Exosome‐based therapies are in the preliminary phases of clinical investigation, with only three clinical trials.

**Conclusion:**

Given the increasing interest in exosome therapy, clinical investigations are expected to increase. This growth will be driven by ongoing advancements in exosome technology, a deeper understanding of their therapeutic potential, and escalating demand for innovative treatment strategies for neurodegenerative diseases.

## Graphical abstract

This review provides a systematic evaluation of stem cell clinical trials (*n* = 94) to treat neurodegenerative diseases, including Alzheimer's disease, Parkinson's disease, amyotrophic lateral sclerosis, and Huntington's disease. It also explores the emerging fields of preclinical and clinical investigations of stem cell‐derived exosome‐based therapies for neurodegenerative diseases.

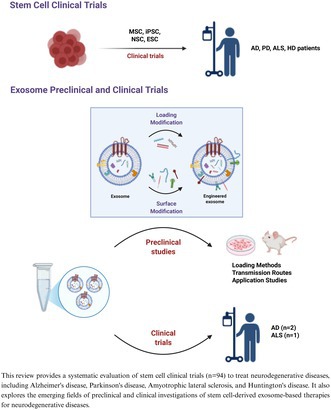

## 1 Introduction

Neurodegenerative diseases (NDs) constitute a group of progressive disorders that primarily impact neurons in the brain and spinal cord. Alzheimer's disease (AD), Parkinson's disease (PD), Amyotrophic lateral sclerosis (ALS), and Huntington's disease (HD) are among the most prevalent NDs, significantly affecting both the individuals diagnosed and their families. These diseases share common features such as progressive neuronal degeneration, which leads to the gradual decline of motor, cognitive, and autonomic functions, although they affect different regions of the nervous system. They are commonly linked to the accumulation of atypical proteins in the brain, such as amyloid plaques and tau tangles in AD, which cause significant brain atrophy through the degeneration of neurons and synapses [1]; alpha‐synuclein (α‐syn) aggregates in PD, which lead to the progressive degeneration of dopaminergic neurons in the substantia nigra, resulting in motor impairments [2]; misfolded proteins like TAR DNA‐binding protein 43 (TDP‐43) or Superoxide dismutase 1 (SOD1) in ALS, which result in the loss of motor neurons and progressive muscle weakness [3]; and the huntingtin (HTT) gene mutation in HD, which results in an abnormally expanded polyglutamine stretch in the HTT protein, leading to toxic intracellular inclusions that disrupt normal cellular functions [4]. As a common characteristic, NDs involve neuroinflammation, mitochondrial dysfunction, and oxidative stress [5], which disrupt the protein clearance systems that normally remove damaged or misfolded proteins, such as autophagy and the ubiquitin‐proteasome system [6], which contribute to neuronal damage. Additionally, they exhibit symptoms steadily deteriorating over time, significantly impairing quality of life and ultimately resulting in mortality. Despite the increasing prevalence of these diseases, effective therapeutic strategies remain limited, and current treatments mostly focus on alleviating symptoms rather than preventing or reversing disease progression. The primary challenge in addressing NDs lies in their complex, multifactorial nature, encompassing genetic, environmental, and cellular factors.

Present pharmacological treatments, such as cholinesterase inhibitors (e.g., donepezil, galantamine, and rivastigmine) and N‐methyl‐D‐aspartate (NMDA) receptor antagonists (e.g., memantine) for AD; dopaminergic medications for PD; riluzole for ALS, and tetrabenazine and deutetrabenazine for chorea in HD, as well as antipsychotics, provide only limited symptomatic relief and do not stop or slow disease progression [7, 8, 9]. Moreover, these medications frequently exhibit adverse side effects and limited effectiveness in later stages of the disease [10]. On the other hand, emerging monoclonal antibody therapies, such as FDA‐approved aducanumab targeting amyloid beta (Aβ), and semorinemab aiming to disrupt tau propagation and aggregation, offer an innovative approach to addressing the pathogenic pathways in AD and tauopathies though their clinical efficacy remains under debate [11, 12]. In the case of HD, strategies such as gene silencing to reduce mutant HTT protein levels are being explored as potential therapies to slow disease progression [13]. Meanwhile, in PD, dopamine replacement therapies, such as levodopa combined with carbidopa, remain the gold standard for symptomatic treatment, although complications like motor fluctuations and dyskinesias often arise with prolonged use [14]. Despite notable progress, obstacles in drug therapy for NDs persist. Many drugs fail to traverse the blood–brain barrier (BBB) effectively, reducing their therapeutic efficacy, and patient‐specific variations in disease pathology complicate treatment responses, requiring the development of personalized medicine approaches to optimize therapeutic outcomes.

A promising novel therapy is gene therapy, which offers several advantages primarily by addressing the genetic causes rather than just alleviating symptoms. It can provide long‐term or even permanent solutions by delivering functional genes to replace defective ones, or by silencing harmful genes. Moreover, gene therapy can potentially target the molecular mechanisms that lead to neurodegeneration, offering a more precise and disease‐modifying treatment than conventional therapies. However, gene therapy shows significant challenges as well, such as delivering therapeutic genes to the brain due to the BBB and ensuring their stable expression in neurons due to the brain's complex structure [15, 16]. Although gene therapy clinical trials for NDs have advanced progressively in recent years, they are in the early to mid‐development phases.

Stem cell therapies, which are the primary focus of this review, have emerged as a promising strategy for NDs. Unlike conventional pharmacological therapies that primarily alleviate symptoms with minimal disease‐modifying effects, stem cell therapies have the ability to repair damaged tissues, replace lost neurons, and modulate neuroinflammation. Despite their significant potential, they face several issues. One major concern is the risk of tumorigenesis, particularly with pluripotent stem cells. Additionally, there are limitations regarding the survival and integration of transplanted cells into host tissue, as well as the risk of uncontrolled differentiation or migration of these cells [17]. Alongside direct stem cell transplantation, an emerging field of research investigates the application of stem cell‐derived exosomes, nanovesicles that transport diverse biomolecules, including proteins, lipids, and RNA, capable of facilitating intercellular communication and impacting mechanisms underlying the disease [18]. Exosomes have multiple advantages over stem cell therapies, such as reduced risk of immunological rejection and tumorigenesis, since they do not include the direct transplantation of viable cells, difficulties associated with cell survival and engraftment may be avoided. Moreover, exosomes are capable of passing through the BBB with greater efficacy and possess the capacity to diminish neuroinflammation, facilitating neuroprotection resulting in cellular repair [19, 20]. Furthermore, exosomes are easier to apply and can be preserved for extended durations at low temperatures without losing their efficacy, rendering them more practical and economical for clinical applications in comparison to stem cells [21].

This review seeks to deliver a comprehensive evaluation of stem cell clinical trials conducted this far on NDs, in conjunction with preclinical and clinical investigations involving the nascent involvement of stem cell‐derived exosomes in AD, PD, ALS, and HD. By examining the latest preclinical studies and clinical trials, we aim to provide a comprehensive overview of how stem cell‐derived exosome therapies could revolutionize the treatments of NDs.

## 2 An Overview of the Stem Cell Therapy in Neurodegenerative Diseases

Stem cell therapies represent a promising advancement in the treatment of NDs due to their potential to repair impaired brain functions and slow disease progression. Various types of stem cells, including neural stem cells (NSCs), mesenchymal stem cells (MSCs), induced pluripotent stem cells (iPSCs), and embryonic stem cells (ESCs), have shown the ability to replace damaged or lost neurons, restore disrupted brain circuits, and integrate into injured brain regions, thereby ameliorating cognitive and motor impairments [22].

**FIGURE 1:**
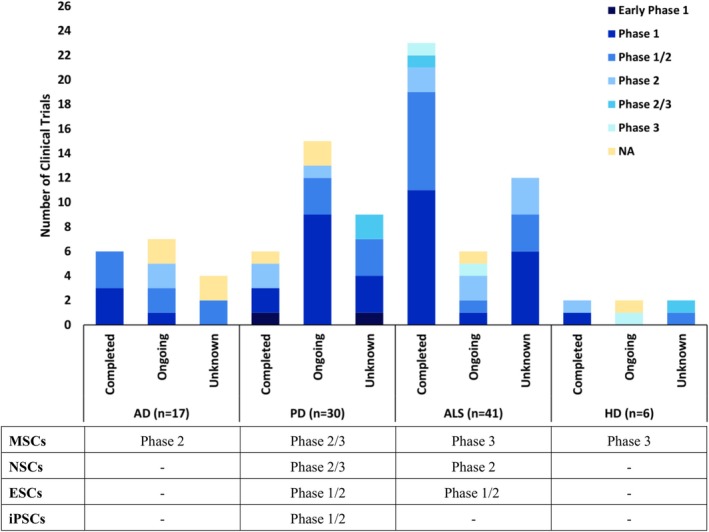
Data illustrating the status of stem cell clinical trials in AD, PD, ALS, and HD categorized by their phases, and the furthest phase each disease has come, as registered in clinicaltrials.gov.

To analyze stem cell clinical trials conducted in NDs, we refined our list to 94 studies. This was achieved by searching ClinicalTrials.gov with the terms “stem cells and Alzheimer's Disease,” “stem cells and Parkinson's Disease,” “stem cells and Amyotrophic Lateral Sclerosis,” and “stem cells and Huntington's Disease” as separate queries, excluding studies that did not involve stem cell‐based cellular therapy in order to obtain a representative dataset. Each study was individually reviewed for inclusion. Trials with recruitment statuses of “suspended,” “terminated,” “withdrawn,” or “no longer available” were excluded, while those labeled “completed,” “not yet recruiting,” “recruiting,” “active, not recruiting,”, “enrolling by invitation,” and “unknown” were included. Studies other than those marked “completed” or “unknown” were grouped as “ongoing.” Additionally, trials were categorized by their clinical phases, including those with unknown phases labeled as NA. The furthest phase each disease has come with stem cell therapy has also been below the graph (Figure 1).

**FIGURE 2:**
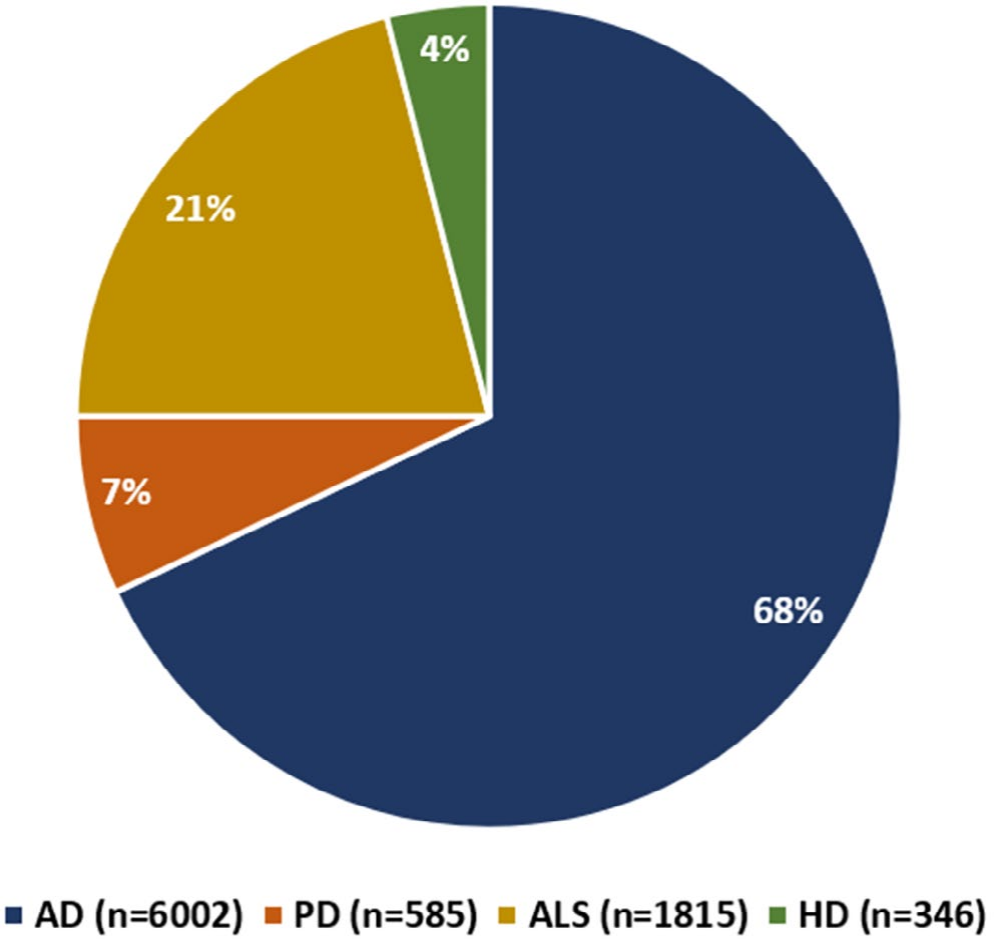
Distribution of participants in clinical trials of stem cell therapy in AD, PD, ALS, and HD, as registered in clinicaltrials.gov.

The initial clinical trials employing stem cells for the treatment of the above‐mentioned NDs began in 2007, starting with ALS, followed by AD and PD in 2011, and much later with HD in 2014. These trials mainly focused on assessing the effectiveness of stem cells in NDs and aimed to evaluate the safety and feasibility of transplanting stem cells into patients through various delivery methods, including intravenous or intrathecal injection, and also direct transplantation into the brain. Out of the 94 clinical trials, if we ignore those with *unknown* study status, there are three Phase 3 trials in total (1 completed and 1 ongoing in ALS, and 1 ongoing in HD), ten Phase 2 trials (2 ongoing in AD, 2 completed and 1 ongoing in PD, 2 completed and 2 ongoing in ALS, 1 completed in HD), and one completed Phase 2/3 trial in ALS. The remaining are mostly Phase 1 and 1/2 trials, and a few early phase trials (Figure 1). In total, more than 8000 participants (patients and healthy controls) have enrolled in stem cell therapy trials across these four diseases. Notably, nearly 70% of these participants were enrolled in AD trials (Figure 2).

NSCs possess the distinctive ability to self‐renew and differentiate into both neurons and glial cells throughout the development of the central nervous system (CNS), making them particularly suitable for treating NDs [23]. NSC transplantation has demonstrated the potential to prevent neurodegeneration and promote regeneration through various mechanisms, including enhancing neuronal plasticity, reducing neuroinflammation, producing neurotrophic factors, and restoring dead cells [24]. However, their use comes with limitations. NSCs are primarily found in particular regions of the adult brain, making it impractical to obtain them from live donors for therapeutic purposes. Additionally, sourcing NSCs from fetal brains raises ethical concerns about the potential exploitation of human fetuses.

**FIGURE 3:**
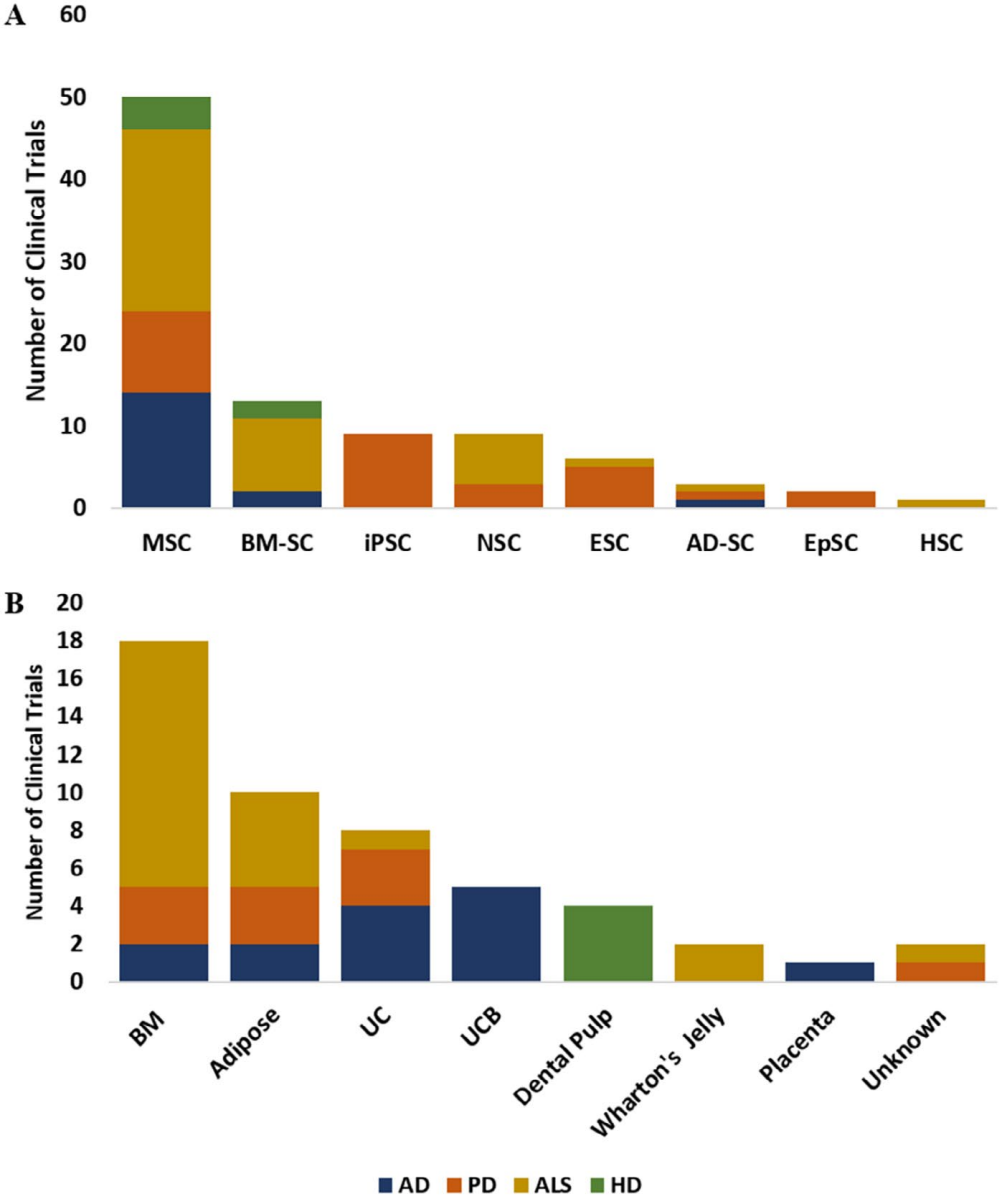
(A) Distribution of types of stem cells used in clinical trials of AD, PD, ALS, and HD, as registered in clinicaltrials.gov. (MSC: Mesenchymal stem cells, BM‐SC: Bone marrow‐derived stem cells, iPS: Induced pluripotent stem cells, NSC: Neural stem cells, ESC: Embryonic stem cells, AD‐SC: Adipose tissue‐derived stem cells, EpSC: Epithelial stem cells, HSC: Hematopoietic stem cells). (B) Sources of isolation of stem cells used in clinical trials of AD, PD, ALS, and HD, as registered in clinicaltrials.gov. (BM: Bone marrow, UC: Umbilical cord, UCB: Umbilical cord blood).

On the other hand, MSCs, often derived from bone marrow, adipose tissue, or umbilical cord tissue, are some of the most widely used stem cells in clinical trials (Figure 3A,B) due to their ability to modify the local microenvironment, support neurogenesis, promote cellular repair, and modulate immune responses, potentially improving cognitive and motor functions. Their immunomodulatory effects and secretion of trophic factors make them attractive for treating NDs, potentially alleviating neuroinflammation and enhancing tissue repair. MSCs are typically regarded as possessing a good safety profile, as they have a reduced propensity for tumor formation and can be used in autologous therapies to mitigate immunological rejection. However, challenges remain regarding the clinical application of MSC therapy. Issues such as cell survival, engraftment and the ability to direct MSC differentiation into neurons or other specific cell types are significant complications that must be overcome before broad clinical implementation [25]. While single MSC transplants are considered safe and do not trigger a significant immunological reaction, frequent administration of MSCs may lead to the development of allo‐antibodies, posing risks of immune complications [26]. Furthermore, MSCs display Major Histocompatibility Complex (MHC) class I, and they may stimulate allogeneic immune responses that cause tissue damage and inflammation recipients [27].

ESCs derived from early‐stage embryos possess the highest potential for differentiation into any cell type. However, their use is highly controversial due to ethical concerns. As a result, clinical trials involving ESCs in NDs are limited (*n* = 6), with one completed, one with unknown status, and three ongoing for PD, and one completed for ALS Figure 3A. In contrast, iPSCs, generated through the reprogramming of adult somatic cells, offer a less controversial alternative. iPSCs maintain comparable pluripotency while significantly reducing ethical concerns. Clinical trials using iPSCs have primarily focused on PD, with nine study records, one completed, one unknown, and seven ongoing. These trials predominantly involve the generation of dopaminergic progenitor cells for direct transplantation into the patients' brains [28]. Recently, there has been a shift toward iPSC and exosome‐based therapies, which provide better control over differentiation processes and minimize the risk of immunological rejection.

**FIGURE 4:**
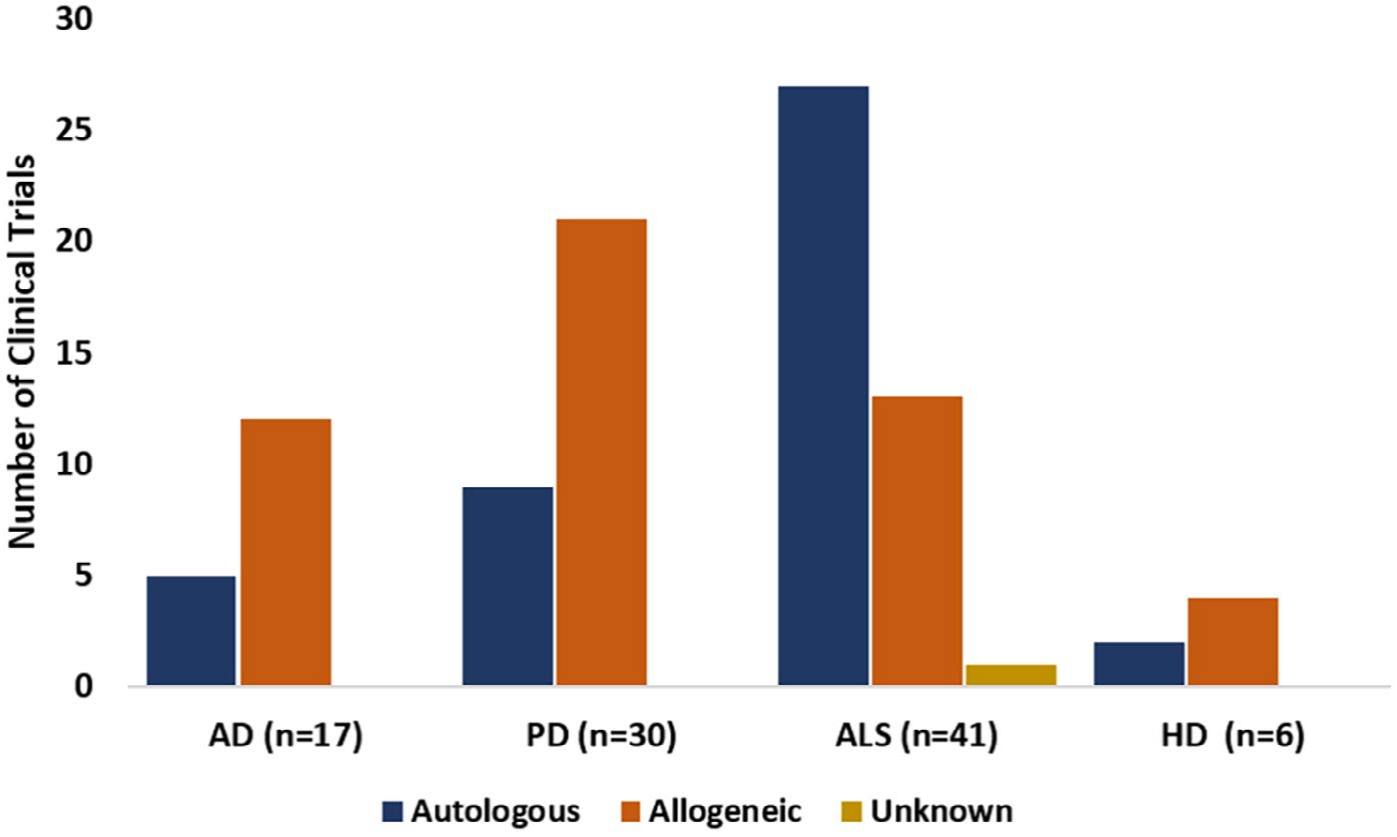
Donor source of the stem cells used in clinical trials of AD, PD, ALS, and HD, as registered in clinicaltrials.gov.

In addition to the source of stem cell isolation, the donor source of stem cells is also an important factor to be considered when designing the most suitable personalized treatment. The choice of donor source of stem cells can either be autologous or allogeneic. Even though allogeneic therapy is mostly preferred in AD and PD, autologous therapy has become prominent in ALS Figure 4. Autologous therapy is less complicated with no risk of rejection as the cells are isolated directly from the patient. However, elderly patients, as well as those with advanced disease stages, have the disadvantage of low quality and even quantity of stem cells. In contrast, allogeneic stem cells tend to be more abundant and healthier, but they now have a risk of immunogenicity resulting in graft vs. host disease [22]. Therefore, the source may be decided based on the disease, patient's condition, and availability of healthy stem cells.

## 3 Exosomes

### 3.1 Characteristics and Biological Functions of Exosomes in the Context of NDs

Exosomes are small vesicles, measuring 30–150 nm in size, which were once considered mere waste disposal units but are now recognized as playing critical roles in various biological processes [29]. Structurally, exosomes consist of a lipid bilayer enriched with sphingomyelin, phosphatidylserine, phosphatidylcholine, and cholesterol, which helps to maintain their stability and allows them to transport bioactive molecules, including therapeutic compounds [30]. They contain proteins associated with the endosomal pathway, such as tetraspanins (CD9, CD63, CD81), heat shock proteins (Hsc70), lysosomal proteins (Lamp2b), and proteins involved in multivesicular body (MVB) formation, like apoptosis‐linked gene 2 (ALG‐2)‐interacting protein X (ALIX) and Tumor susceptibility gene 101 (Tsg101) [31]. These proteins help distinguish exosomes from other extracellular vesicles and contribute to their roles in antigen presentation, cellular uptake, and release. Exosomes also carry RNA, including microRNAs (miRNAs), long noncoding RNAs (lncRNAs), and circular RNAs (circRNAs), which are essential for regulating gene expression in recipient cells. miRNAs, in particular, are abundant and play an important role in intercellular communication, influencing the phenotype of target cells. Due to circRNA's stability, they are also gaining attention as potential biomarkers for diagnostics [32].

Exosomes are vital in maintaining homeostasis and facilitating intercellular communication in the CNS, both of which are essential for optimal brain function. They participate in key biological processes, including synaptic plasticity, neurogenesis, and myelination, as well as neuronal protection and regeneration following injury or disease. They promote neurogenesis by influencing stem cell activity, enhance synaptic activity and plasticity to support learning and memory, aid in the formation and maintenance of myelin sheaths for efficient neural signaling, and contribute to neuronal recovery under pathological conditions [33, 34].

Exosomes can be isolated from nearly all body fluids, such as blood, urine, saliva, and cerebrospinal fluid. Their molecular content reflects their cellular origin, impacting various physiological and pathological processes [19]. Exosomes derived from MSCs are the most common type of exosomes sourced from stem cells to be used in NDs. These exosomes exhibit specific surface markers, such as CD29, CD44, CD73, CD90, and CD105, but lack markers like CD11b, CD34, and CD45, distinguishing them from other cell types. They also express universal exosome markers, including CD9, CD63, CD81, and ALIX, and carry bioactive lipids and miRNAs like miR‐26b and miR‐206, which contribute to their therapeutic effects [35]. MSC‐derived exosomes are not only immunomodulators but also effective agents in tissue repair and homeostasis. They carry growth factors like vascular endothelial growth factor (VEGF) and transforming growth factor‐beta (TGF‐β) that stimulate angiogenesis, enhancing wound healing and vascular regeneration [36]. Their miRNA cargo, such as miR‐21 and miR‐17‐92 clusters, promotes anti‐apoptotic pathways by inhibiting phosphatase and tensin homologue (PTEN) and activating protein kinase B (Akt) signaling, thereby enhancing cell survival. Additionally, they play a role in neurogenesis by delivering miRNAs like miR‐124, which facilitate neural precursor differentiation. These exosomes also help regulate oxidative stress by lowering reactive oxygen species (ROS) levels, offering protection against neurodegeneration [37].

Other types of exosomes, such as those derived from NSCs, also play significant roles in NDs. They express neuron‐derived neurotrophic factor (NDNF) and neurogenic miRNAs, such as miR‐9 and miR‐124. These molecules are crucial for promoting neuroprotection, neuronal differentiation, and synaptic plasticity, making NSC‐derived exosomes particularly effective in supporting neural regeneration and repair [38]. In addition to neurogenesis, they enhance synaptic connectivity and regulate neurotransmission, which are crucial for cognitive functions. These exosomes carry neurotrophic factors like brain‐derived neurotrophic factor (BDNF) that promote neuronal growth and repair [39]. Their miRNA cargo, specifically miR‐124 and miR‐132, supports neuronal differentiation and inhibits pro‐apoptotic proteins like Bcl‐2‐associated X protein (Bax) [40]. They also reduce neuroinflammation by balancing microglial activation states, potentially slowing neurodegenerative processes [41]. Importantly, their ability to traverse the BBB makes them promising therapeutics for targeted therapies in NDs.

### 3.2 Exosome Biogenesis

**FIGURE 5:**
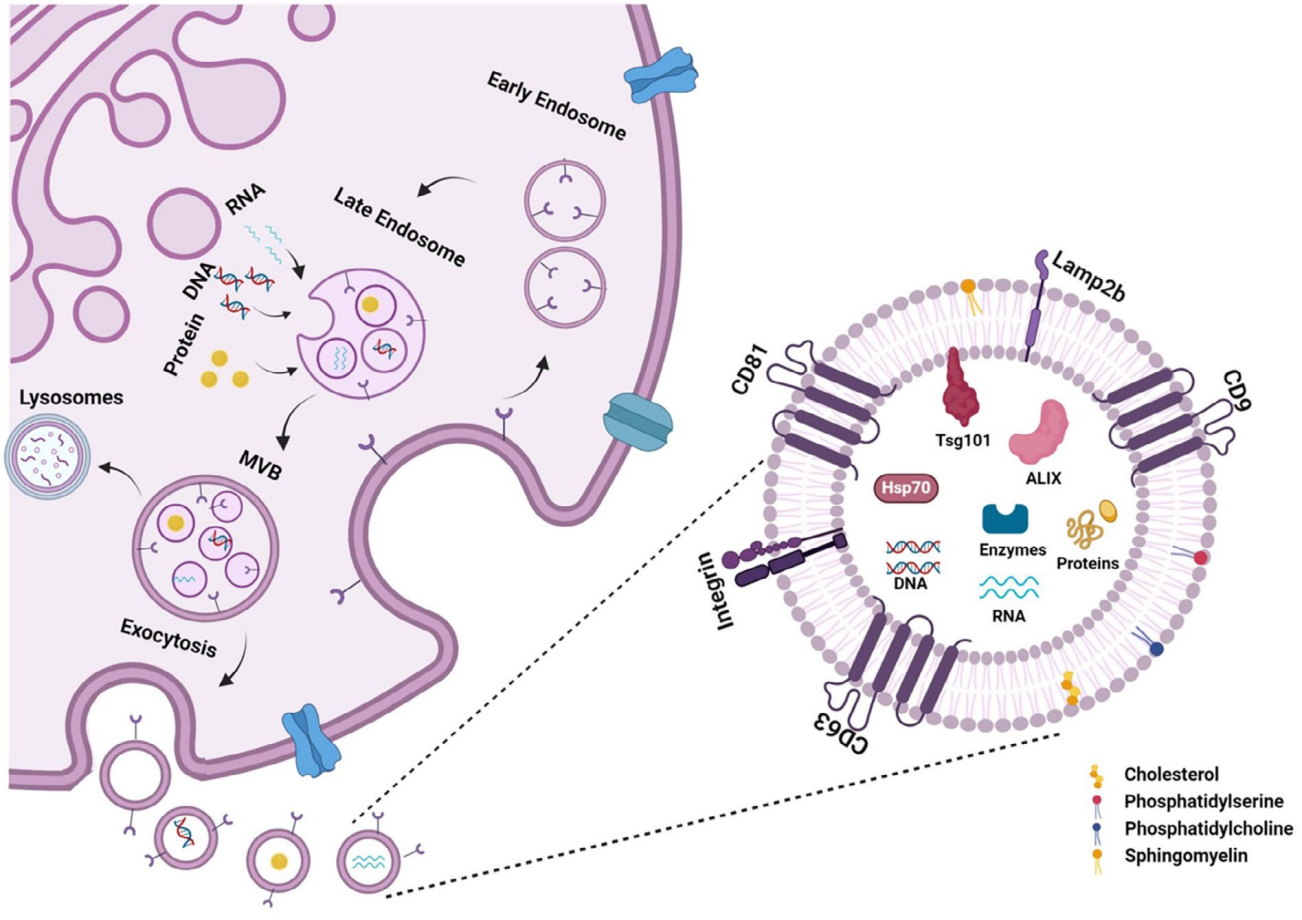
Biogenesis and molecular composition of exosomes. Exosomes are formed during the maturation of early endosomes into late endosomes and multivesicular bodies (MVBs), which fuse with the plasma membrane to release exosomes into the extracellular space. Their molecular components include membrane lipids (cholesterol, phosphatidylserine, phosphatidylcholine, and sphingomyelin), transmembrane proteins (CD81, CD63, CD9, and Lamp2b), cytosolic proteins (ALIX, Tsg101, and Hsp70), and cargo such as RNA, DNA, enzymes, and other proteins, highlighting the diverse molecular content of exosomes critical for intercellular communication.

As depicted in Figure 5, exosome biogenesis initiates within the endosomal system of eukaryotic cells, beginning with the invagination of the cytoplasmic membrane to form early endosomes. These early endosomes subsequently mature into late endosomes, referred to as multivesicular bodies (MVBs). During this maturation, intraluminal vesicles (ILVs) are produced through inward budding of the endosomal membrane into the MVB lumen. The MVBs can either fuse with lysosomes or autophagosomes for degradation or transport to the plasma membrane, where they merge and release ILVs into the extracellular environment as exosomes [42].

Exosome biogenesis can occur through both Endosomal Sorting Complex Required for Transport (ESCRT)‐dependent and ESCRT‐independent mechanisms:

#### 3.2.1 ESCRT‐Dependent Mechanism

This pathway involves a series of four protein complexes (ESCRT‐0, ‐I, ‐II, and ‐III) along with associated proteins. ESCRT‐0, composed of hepatocyte growth factor–regulated tyrosine kinase substrate (HRS) and signal transducing adapter molecule (STAM), plays a pivotal role in identifying and sequestering ubiquitinated proteins through ubiquitin‐binding domains that are targeted for inclusion in ILVs. ESCRT‐I and ESCRT‐II are responsible for initiating membrane invagination, while ESCRT‐III forms a helical structure that constricts the neck of the budding vesicle. The hexameric AAA ATPase Vps4 (Vacuolar Protein Sorting‐Associated Protein 4) facilitates the final fission, releasing ILVs into the MVB lumen. Accessory proteins such as ALIX and Vps4 are essential for recycling the ESCRT machinery and supporting the overall vesicle formation process [43].

#### 3.2.2 ESCRT‐Independent Mechanism

Exosome formation can also occur without the involvement of ESCRT proteins, utilizing alternative molecules such as lipids and tetraspanins. Specific lipids, including ceramide, cholesterol, and phosphatidic acid, are crucial for promoting membrane invagination and facilitating vesicle formation. Tetraspanins enhance cargo sorting by creating specialized membrane microdomains that assist in vesicle budding. Additionally, heat shock proteins (HSP) recognize particular motifs in cytosolic proteins, directing them into ILVs [44].

Both pathways are often active simultaneously, with different exosome subtypes forming through distinct mechanisms. The secretion of exosomes is regulated by small Rab GTPases, including Rab27a, Rab27b, Rab35, and Rab7, which coordinate vesicle transport to the plasma membrane [45]. Furthermore, external stimuli such as hypoxia or inflammation can significantly influence the composition and functional properties of exosomes, particularly in MSCs [46].

## 4 Therapeutic Potential of Exosomes in Neurodegenerative Diseases

The therapeutic potential of exosomes in NDs arises from their capacity to deliver neuroprotective and regenerative signals, cross the BBB, and minimize the risks associated with stem cell therapy [18]. According to preclinical studies, exosomes have been shown to deliver neuroprotective drugs, diminish neuroinflammation, and facilitate neuronal regeneration in animal models, underscoring their medicinal potential [47, 48, 49]. Moreover, engineered exosomes, modified to transport certain pharmaceuticals or genetic material, demonstrate potential in improving treatment effectiveness. These advancements place exosomes in the front of research focused on identifying more effective and targeted therapies for these severe conditions [46]. The subsequent sections will provide a detailed discussion of preclinical and clinical trials completed to date for AD, PD, ALS, and HD along with studies employing engineered exosomes.

### 4.1 Preclinical Investigations Into the Therapeutic Potential of Stem Cell‐Derived Exosomes in NDs

#### 4.1.1 Alzheimer's Disease

MSC‐derived exosomes are increasingly recognized as a promising therapeutic strategy for AD because of their potent anti‐inflammatory, neuroprotective, and neuroregenerative properties. They are chosen over other stem cells, such as iPSCs and NSCs, for AD treatment due to their potent immunomodulatory effects, which help mitigate neuroinflammation, a key factor in AD progression [50]. These exosomes, rich in neurotrophic factors, proteins, and miRNAs, help mitigate neuroinflammation, one of the primary contributors to AD pathology [51]. They contain exosomal cargo, including miRNAs, which are essential in controlling neural remodeling, angiogenesis, and neurogenesis, processes essential for mitigating AD‐related cognitive decline [52]. In vivo investigations have also revealed the potential of exosomes produced from MSCs in AD models. A study demonstrated that MSC‐derived exosomes ameliorated AD‐like characteristics in a preclinical mouse model, decreasing neuroinflammation and enhancing cognitive performance [53]. These findings are supported by research showing that MSC‐derived exosomes stimulate neurogenesis, enhance cognitive function recovery, and improve neuronal differentiation in mouse models of AD [54].

NSC‐derived exosomes deliver neuroprotective molecules that promote neuronal survival and reduce neuroinflammation. A research group reported that these exosomes stimulated neurogenesis, enhanced memory and learning, and reduced p‐tau expression levels and Aβ generation [55]. Exosomes derived from glial cells, including astrocytes and microglia, are emerging as additional therapeutic tools in AD. Astrocyte‐derived exosomes carry neuroprotective molecules that aid neuronal survival and modulate synaptic activity and neuroinflammation. Furthermore, microglia‐derived exosomes can influence AD progression by modulating inflammatory responses. To illustrate, elevated levels of miR‐124‐3p in microglial exosomes supported anti‐inflammatory M2 polarization, reduced neuroinflammation, and enhanced neurite extension [55, 56].

The infusion of glycosphingolipid‐containing exosomes into the brains of Amyloid precursor protein (APP) transgenic mice reduced Aβ levels and alleviated amyloid‐related pathologies, suggesting their potential as messengers for Aβ clearance [57]. Thus, exosomes underscore the complexity of their function in AD and highlight the need for targeted strategies to harness their therapeutic potential while minimizing adverse effects.

#### 4.1.2 Parkinson's Disease

The therapeutic potential of exosomes in PD has been extensively investigated in preclinical studies, revealing diverse mechanisms and significant promises for neuroprotection both in vitro and in vivo models. These models have highlighted the efficacy of MSC and NSC‐derived exosomes in reducing pathological features of PD. MSC‐derived exosomes, enriched with neurotrophic and anti‐inflammatory factors, have been shown to reduce α‐syn aggregation, promote autophagy, enhance dopaminergic neuron survival, and improve motor deficits. For instance, they improved neurovascular function through angiogenesis in brain microvascular endothelial cells and inhibited microglial activation and reduced neuroinflammation [41, 58]. Similarly, another study demonstrated that MSC‐derived exosomes enhanced neuronal repair by promoting autophagy [59]. Beyond single‐agent applications, novel combinations have emerged, and MSC‐derived exosomes improved neuronal survival and motor function by surpassing L‐Dopa in a rotenone‐induced PD model [60]. Furthermore, NSC‐derived exosomes demonstrated significant neuroprotective effects by reducing oxidative stress and inflammation while preserving dopaminergic neurons in in vitro and in vivo 6‐OHDA‐induced PD models [61].

#### 4.1.3 Amyloid Lateral Sclerosis

Exosomes derived from AD‐SCs have been investigated in cellular models of ALS. Introducing healthy human AD‐SCs to murine SOD1‐G93A, NSCs was found to slow down SOD1 aggregation and ameliorate mitochondrial activity [62]. In addition, exosomes isolated from murine AD‐SCs were applied to NSC‐34 cells overexpressing SOD1‐G93A, SOD1‐G37R, and SOD‐1A4V, which were exposed to oxidative stress induced by hydrogen peroxide. Hence, this treatment diminished oxidative damage, restored mitochondrial activity, and ameliorated cell survival [63, 64]. Another research investigated neuroprotection using exosomes derived from AD‐SCs in an in vivo ALS model. The researchers also tested both intravenous and intranasal administration methods for AD‐SC exosomes in vivo in SOD1‐G93A mice. Using ultra‐small superparamagnetic iron oxide nanoparticles to label the exosomes, MRI results demonstrated their ability to cross BBB and localize in the brain. Mice treated with exosomes exhibited enhanced motor functions compared to the placebo group. Furthermore, the exosomes demonstrated an innate capability to target the ALS‐affected regions within the brain. Therefore, these results provided important insights into the potential application of AD‐SC‐derived exosomes as a therapeutic option for ALS in humans [65]. Exosomes derived from MSC were evaluated for their effects on ALS (SOD1‐G93A transgenic) and wild‐type primary motor neurons. It was shown that the MSC exosomes had beneficial effects on neurite development and morphology under normal and stressed conditions, highlighting their potential for more preclinical research and the development of clinical treatments. Furthermore, gene expression analysis revealed the presence of several antioxidants and anti‐inflammatory genes within the MSC exosomes [66].

#### 4.1.4 Huntington's Disease

Exosomes have emerged as critical mediators of intercellular communication, particularly in the transport of regulatory molecules like miRNAs, which play essential roles in gene regulation [67]. They have been proposed as delivery vehicles, as they can cross the BBB and serve as an ideal means of transporting small therapeutic molecules like miRNAs to the CNS. In HD, miRNA‐124, which typically reduces the RE‐1 silencing transcription factor (REST) gene to promote BDNF production, is downregulated, leading to increased levels of REST and BDNF repression [68]. Given miR‐124's role in adult neurogenesis, particularly in the subventricular zone, its delivery to affected regions is being explored as a potential therapy. A preclinical study demonstrated that exosomes derived from HEK‐293 cells overexpressing miR‐124, when administered to an R6/2 HD mouse model, successfully escalated miR‐124 levels and reduced REST protein. However, no significant clinical improvements in HD pathology were observed, likely due to the low miR‐124 content in the exosomes. Exosome‐based therapies are highly promising due to the versatile roles exosomes play in intercellular communication and therapeutic delivery. Specific miRNAs, such as miR‐214, miR‐150, miR‐9, and miR‐22, have been identified as having potential applications in future therapeutic strategies for HD [69]. In further research, exosomes loaded with small interfering RNAs (siRNAs) targeting the HTT gene were shown to reduce mHTT expression in HD mouse models [70]. Moreover, exosomes derived from AD‐SCs have shown neuroprotective effects, including a reduction in mHTT aggregates and an increase in proteins associated with cellular protection [71]. The literature on the use of exosomes derived from stem cells, such as MSCs, NSCs, or iPSCs, in HD therapy is currently very limited. While stem cell‐derived exosomes have shown therapeutic potential in various neurodegenerative diseases due to their neuroprotective properties [19], specific preclinical studies demonstrating their efficacy in HD models are scarce.

The sources, defining characteristics, and outcomes of the stem cell‐derived exosomes used in in vitro and in vivo research conducted for AD, PD, ALS, and HD are summarized in Table 1.

**TABLE 1:** Sources, characteristics, and outcomes of therapeutic exosomes in NDs.

ND	Exosome Sources	Model Type	Description	Outcomes	Ref.
AD	UC‐MSC	In vivo	Exosomes with anti‐inflammatory and neuroprotective effects	↑ Neuroprotection ↓ Aβ aggregation ↓ Oxidative stress	92
	NSC	In vitro	Exosomes enriched with neural‐specific proteins and RNAs	↑ Neurogenesis ↑ Memory and learning ↓ Neuroinflammation ↓ p‐tau and Aβ generation	55
PD	MSC	In vitro and in vivo	Exosomes carrying neurotrophic factors and anti‐inflammatory molecules	↑ Dopaminergic neuron survival ↓ α‐syn aggregation ↓ Neuroinflammation	58–60
	NSC	In vitro and in vivo	Exosomes promote dopaminergic differentiation and neuroprotection.	↑ Dopaminergic function ↓ Motor deficits ↓ Oxidative stress	61
ALS	MSC	In vitro	Exosomes with anti‐inflammatory and immunomodulatory properties	↑ Cellular survival ↑ Neuroprotection ↑ Neurite development ↓ Motor neuron inflammation	66
	AD‐SC	In vitro and in vivo	Exosomes with neuroprotective effects	↑ Neuroprotection ↑ Brain localization ↑ Motor functions ↑ Mitochondrial activity ↑ Cell survival ⊣ Apoptosis ↓ ROS	62–65
HD	HEK‐293 cells	In vivo	Exosomes engineered to overexpress miR‐124	↑ miR‐124 levels ↓ REST protein	69
	AD‐SC	In vitro	Exosomes with neuroprotective effects	↑ Neuroprotection ↑ Cellular protection ↓ mHTT aggregates	71

### 4.2 Clinical Trials for Stem Cell‐Derived Exosomes in NDs

It is widely recognized that exosomes are a promising option for treating many diseases. Plenty of preclinical studies have shown the benefits of exosomes in treating a range of conditions. Lately, exosomes have been explored in clinical trials, particularly those obtained from human stem cells. Besides, the primary uses of exosomes in clinical applications include serving as biomarkers, providing exosome‐based therapies, and aiding drug delivery systems [72]. Currently, clinical trials investigating the use of exosomes in NDs remain scarce, reflecting the novelty of this field. Despite their potential as innovative therapeutic tools, exosome‐based treatments are still in the early stages of exploration, with much to learn about their mechanisms and applications.

A recruiting clinical trial, which has completed Phase 1, seeks to assess the safety and preliminary effectiveness of nasal drop exosomes isolated from human UC‐MSC in its Phase 2 stage for the treatment of ALS (ClinicalTrials.gov identifier: NCT06598202). A new study, which has not yet begun recruitment, aims to investigate the impact of an extended program combining aerobic exercise and cognitive training on both cognitive abilities and biomarkers (blood neuro‐exosomal synaptic proteins) in patients at high risk of AD and with low levels of specific biomarkers. By analyzing biomarkers such as growth associated protein 43 (GAP43), neurogranin, synaptosome‐associated protein 25 (SNAP25), and synaptotagmin within exosomes circulating in the patients' blood, researchers will be able to identify early signs of AD before its clinical onset (ClinicalTrials.gov identifier: NCT05163626). Furthermore, another study was conducted to investigate the safety and efficiency of exosomes obtained from AD‐MSC for the treatment of AD. In this clinical trial, exosomes were intranasally administered, which showed improved cognition in patients (ClinicalTrials.gov identifier: NCT04388982).

Due to the absence of exosome‐based therapies for some NDs, researchers have nevertheless explored the potential of exosomes as diagnostic tools. For instance, one of the observational studies examined the factors contributing to neurocognitive disorders, with a particular focus on AD, by analyzing blood components, including exosomes and their associated biomarkers (ClinicalTrials.gov identifier: NCT03275363). Another study was conducted to explore potential biomarkers for PD through the analysis of urine exosomes. Urine samples were collected from the patients to examine the presence of specific biomarkers within the exosomes. It was identified that Leucine‐rich repeat kinase 2 (LRRK2) and other proteins were linked to the pathophysiology of PD (ClinicalTrials.gov identifier: NCT01860118).

While there are a few clinical trials investigating exosome therapy for AD and ALS, there are no clinical trials specifically targeting PD and HD (Table 2). As exosome therapy represents a novel and promising treatment approach, further research and clinical exploration are essential to evaluate its potential in addressing the complexities of NDs. In the preliminary phases of development, exosome therapies exhibit significant potential for enhancing the treatment of NDs by providing a safer, more effective, and more accessible alternative to stem cell‐based interventions [73, 74], Table 3.

**TABLE 2:** Clinical trials of exosome therapy in NDs.

ND	Clinical Trial ID	Exosome Source	Status	Study Type
AD	NCT05163626	Blood	Not yet recruiting	Not Applicable
	NCT04388982	AD‐MSC	Unknown	Phase 1/2
ALS	NCT06598202	UC‐MSC	Recruiting	Phase 1/2

**TABLE 3:** Loading modifications of exosomes in NDs according to pathology, disease model and their outcomes.

Loading Modification	Molecule	ND	Model Type	Outcomes	Ref.
Nucleic Acid‐Loaded Exosomes	mRNA	PD	In vitro and in vivo	↓ Neurotoxicity ↓ Neuroinflammation	78
	siRNA	PD	In vivo	↓ α‐syn aggregation ↓ Neuroinflammation	79
			In vitro and in vivo	↑ TH ↓ α‐syn aggregation ↓ Neuronal death	80
		HD	In vivo	↑ Animal behavior ↓ mHTT expression	70
	miRNA	PD	In vivo	↑ Proliferation ⊣ Autophagy ⊣ Pyroptosis	82
		PD	In vitro and in vivo	↓ Neuroinflammation ↓ Oxidative stress	83
		PD	In vitro and in vivo	↓ Dopaminergic neurons ↑ Motor function ↓ Oxidative stress	84
	ASO	PD	In vivo	↑ TH‐positive dopamine neurons ↑ Motor function ↓ α‐syn expression	85
Protein‐Loaded Exosomes	Neprilysin	AD	In vivo	↑ Pro‐inflammatory genes ↓ Aβ1–40 level ↓ Neuroinflammation	75
	Catalase	PD	In vivo and in vitro	↑ Neuroprotection ↓ Oxidative stress	86
	BDNF	PD	In vitro and in vivo	↑ BBB penetration ↑ Neuronal survival ↑ Antioxidant defense ⊣ Apoptosis	89
Antioxidant‐Loaded Exosomes	Curcumin	PD	In vivo	↑ Dopamine level ↑ Movement ↓ α‐syn aggregation	48
	Resveratrol	AD	In vivo and in vitro	↑ Cognition ↑ Biocompatibility ⊣ Aβ1–42 aggregation	91
	Quercetin	AD	In vivo	↑ Brain targeting ↑ Bioavailability ↓ Tau hyperphosphorylation	49
	Oleuropein & Baicalein	PD	In vitro	↑ Cellular uptake ↑ Integrity of BBB ↓ α‐syn fibrillation	47

### 4.3 Engineered Exosomes

#### 4.3.1 Biochemical Surface Modifications

Attaching specific ligands to the surface of exosomes can enhance their interaction with cellular target sites. Altering their surface could be a viable strategy to ameliorate their delivery across the BBB, facilitating targeted delivery to brain cells and ameliorating their stability [75, 76]. These modifications include noncovalent and covalent interactions, electrostatic interactions, and the use of genetic engineering techniques. Folic acid (FA), a widely recognized small molecule, enhances the uptake of exosomes through endocytosis into the brain, owing to the high expression of its receptors on the BBB. Besides, exosomes can be modified via the carboxyl group on FA, which can be linked to the exosome surface via electrostatic interaction [76]. RVG29, a 29 amino acid neuro‐specific peptide derived from the rabies virus glycoprotein (RVG), was chemically modified to MSC‐derived exosomes and enhanced the delivery of exosomes by directing them specifically to the cortex and hippocampus in AD mice. Consequently, improved cognition, decreased Aβ levels, and regulated inflammatory cytokines were observed [77]. RVG was also displayed on the surface of MSC‐derived exosomes, allowing the interaction between this peptide and acetylcholine receptor. This facilitated the interaction between the peptide and acetylcholine receptors, promoting the uptake of exosomes by dopaminergic neurons in an in vivo PD model. P‐selectin glycoprotein was also attached to exosomes to facilitate their interaction with specific receptors on the BBB. This interaction enabled the exosomes to deliver therapeutic agents to the brain. By attaching P‐selectin, the exosomes were better equipped to recognize and bind to endothelial cells in the BBB, enhancing the efficiency of drug delivery to target brain tissues in PD. Therefore, the attachment of RVG and P‐selectin peptides to MSC‐derived exosomes was used for brain‐targeted delivery through intranasal administration, offering a noninvasive approach to bypass the BBB and efficiently transport therapeutic agents to the brain for treating NDs [48].

#### 4.3.2 Loading Modifications

**FIGURE 6:**
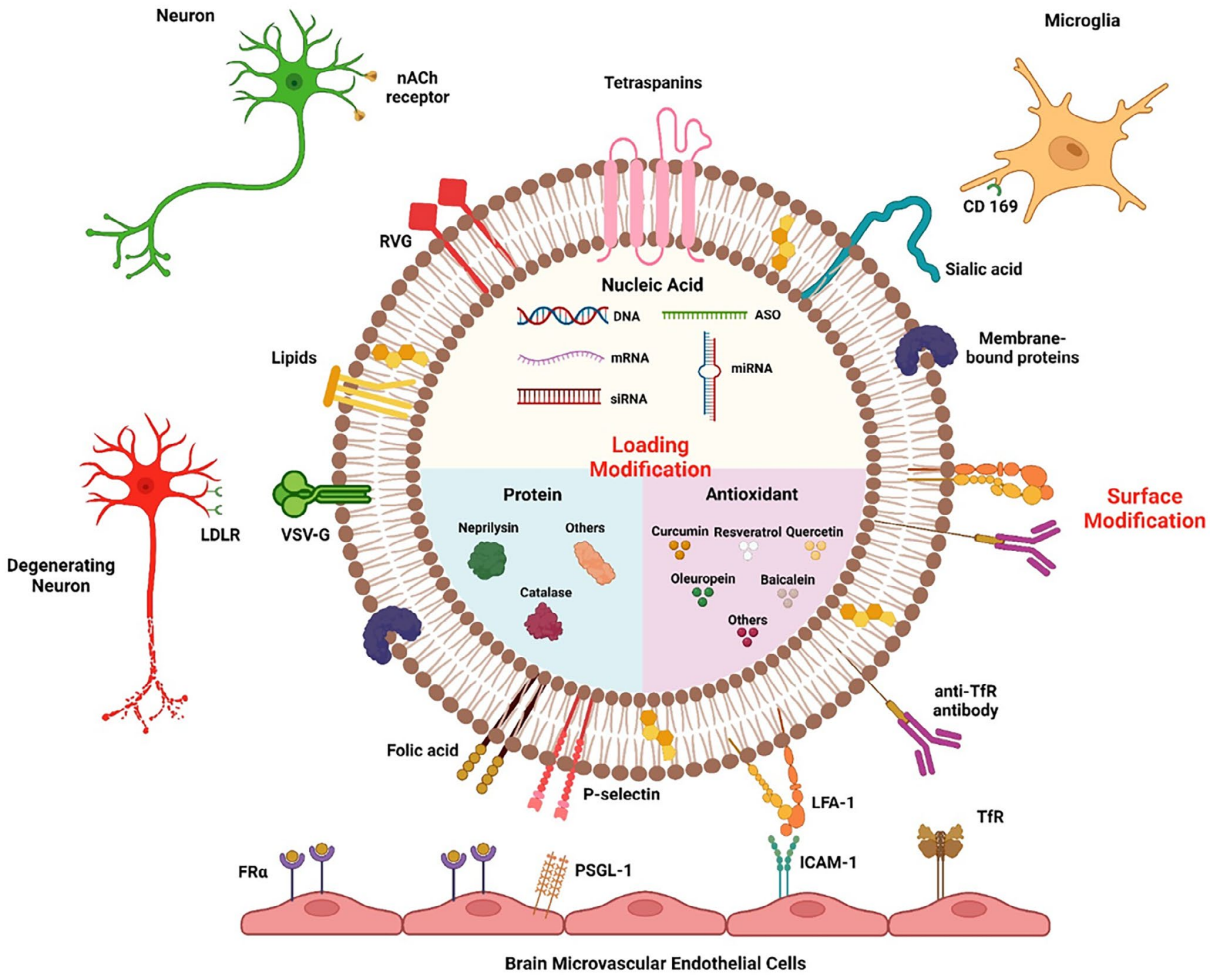
Advancements in exosome modification: Strategies for cargo loading and surface modifications. LDLR: Low density lipoprotein receptor, VSV‐G: Vesicular stomatitis virus glycoprotein, PSGL‐1: P‐selectin glycoprotein ligand‐1, LFA‐1: Lymphocyte function‐associated antigen 1, ICAM‐1: Intercellular Adhesion Molecule 1, TfR: Transferrin receptor, CD 169: Sialic acid‐binding Ig‐like lectin 1, nACh receptor: Nicotinic acetylcholine receptor.

Diverse therapeutic molecules can be incorporated into exosomes without altering their architecture. Exosomes enriched in such molecules can extend their presence in the bloodstream, diminish their potential to trigger immune responses, and consequently enhance the effectiveness of drug treatments (Figure 6). Briefly, this approach could have broader applications for treating various NDs.

##### 4.3.2.1 Nucleic Acid‐Loaded Exosomes

The delivery of therapeutic catalase mRNA through engineered exosomes reduced neurotoxicity and neuroinflammation in both in vitro and in vivo models of PD, highlighting the promising potential of exosomes for delivering RNAs [78]. Moreover, the systematic administration of dendritic cell‐derived exosomes enriched in α‐syn siRNA to S129D α‐syn transgenic mice reduced the overall expression levels of human α‐syn protein across the brain, covering critical regions such as the midbrain, cortex, and striatum. These areas are significantly impacted by α‐syn accumulation at various phases of PD pathology. Furthermore, engineered exosomes have been utilized to mitigate neuroinflammation associated with PD [79]. MSC‐derived exosomes loaded with siRNA targeting fat mass and obesity‐associated protein (FTO) demonstrated a reduction in neuronal damage through m6A‐dependent regulation of ataxia telangiectasia mutated mRNA, ultimately protecting dopaminergic neurons [80]. In addition, the administration of siRNA‐loaded exosomes in mouse models of HD effectively decreased mHTT mRNA and protein expression levels in the brain [70, 81]. Moreover, the use of AD‐MSC exosomes enriched with miR‐188‐3p has shown effectiveness in inhibiting both autophagy and pyroptosis in MPTP‐induced mouse models as well as in MPP + ‐induced MN9D neuronal cells. This study enhanced cell proliferation rates following the treatment with miR‐188‐3p‐enriched exosomes. This underscores the therapeutic potential of miR‐188‐3p, introducing the innovative application of miRNA‐enriched exosomes as a new therapeutic approach for PD [82]. A research group further highlighted the antioxidant effects of miR‐100a‐5p‐enriched exosomes derived from trophoblast stage MSCs, which reduced oxidative stress and neuroinflammation by regulating the NADPH oxidase‐4 (Nox4)/ROS/nuclear factor erythroid 2‐related factor 2 (Nrf2) signaling pathway in MPTP‐treated mice and MN9D cell‐based PD models [83]. Exosomes enriched with miR‐23b‐3p also showed therapeutic effects by modulating the Wnt signaling pathway and promoting autophagy [84]. Furthermore, antisense oligonucleotides (ASOs) are the predominant approach in gene therapy, functioning through RNase H‐mediated degradation of specific RNA sequences. A previous study showed that ASO4 emerged as the most potent antisense oligonucleotide sequence, leading to a substantial decrease in α‐syn levels. Thereby, loading ASO4 into exosomes obtained from BM‐MSCs decreased α‐syn expression and markedly enhanced motor function in the PD mouse model [85].

##### 4.3.2.2 Protein‐Loaded Exosomes

Incorporating therapeutic molecules is often necessary to obtain therapeutic exosomes [86]. Neprilysin is a vital metalloproteinase enzyme essential for Aβ degradation in AD. Its upregulation increases Aβ cleavage and decreases Aβ plaque formation [87]. However, neprilysin cannot cross the BBB, necessitating the development of strategies for its delivery to the brain [88]. Exosomes isolated from AD‐SCs were loaded with neprilysin that specifically targeted and degraded Aβ, which is crucial in AD progression. They could migrate and accumulate in the hippocampus of the brain, significantly lowering levels of Aβ1–40 and diminishing neuroinflammation [75]. Catalase is a crucial antioxidant enzyme that reduces oxidative stress within cells by breaking down hydrogen peroxide into water and oxygen. This process protects the cells from oxidative damage and ensures the maintenance of cellular health and function in PD [86]. Raw 264.7 macrophage‐derived exosomes enriched in catalase effectively targeted neurons and microglial cells in the mouse brain, resulting in neuroprotection in an in vivo PD model. Indeed, exosomes enriched with drugs could represent an advanced drug delivery system that merges the benefits of nanoparticle dimensions with nontoxic properties, substantial drug loading capabilities, and minimal immune response elicitation [86]. In 6‐OHDA‐induced PD models, exosomes derived from human UC‐MSCs loaded with BDNF improved motor function and neuronal survival in vivo by crossing the BBB. Additionally, they enhanced antioxidant defenses by activating the Nrf2 signaling pathway [89].

#### 4.3.3 Antioxidant‐Loaded Exosomes

Antioxidants can be loaded into exosomes to increase their therapeutic efficiencies. Curcumin, a potent polyphenolic compound extracted from the rhizomes of *Curcuma longa* , exhibits anti‐inflammatory and antioxidant properties. Studies have highlighted its beneficial impact in treating NDs by mitigating neuroinflammation and oxidative stress in conditions like AD and inhibiting the misfolding or aggregation of proteins like α‐syn in PD [90]. A study has demonstrated that small neuroprotective compounds such as curcumin prevented the buildup of α‐syn, aiding in eliminating the toxic aggregates through autophagy and diminishing their harmful effects on dopaminergic neurons. It has been observed that MSC‐derived exosomes enriched in curcumin reduced α‐syn aggregates and decreased their neurotoxic effects. These exosomes also played a crucial role in alleviating neuroinflammation by lowering α‐syn levels and enhancing exosome activity. Consequently, mice had a notable enhancement in motor function and coordination. By delivering drugs directly into the cytoplasm of dopaminergic neurons through membrane fusion, the exosomes provided a comprehensive treatment for PD, addressing its multifaceted pathologies [48]. The efficacy of Olesoxime‐Resveratrol‐loaded exosomes reduced Aβ1‐42 levels and significantly enhanced learning and memory functions of the mice. Building on these findings, they present a promising and effective approach for AD treatment [91, 92]. Another research demonstrated that exosomes derived from blood plasma enriched with quercetin facilitated more effective targeting of the antioxidant to the brain and markedly increased its bioavailability [92]. Moreover, quercetin‐loaded exosomes were more efficient than unencapsulated quercetin in alleviating AD symptoms by suppressing the phosphorylation of Tau through cyclin‐dependent kinase 5 inhibition and diminishing the accumulation of insoluble neurofibrillary tangles [49]. A recent research combined exosomes from human UC‐MSCs with nanoliposomes loaded with baicalein and oleuropein, diminishing α‐syn fibrillation. This study not only reduced the pathogenic effects of α‐syn but also ameliorated the uptake of therapeutic agents into the cells [47].

## 5 Conclusion and Future Prospects

This review emphasizes the progress in stem cell therapy for NDs, including AD, PD, ALS, and HD. Despite notable advancements, especially in early‐phase clinical trials, the scarcity of late‐stage trials highlights the necessity for additional investigations. The emergence of stem cell‐derived exosome‐based therapeutics provides a persuasive alternative to direct stem cell transplantation, facilitating less invasive methods for the delivery of therapeutic molecules across the BBB.

Exosomes, especially those originating from MSCs, have exhibited considerable preclinical efficacy in alleviating neuroinflammation, diminishing oxidative stress, and facilitating neuronal regeneration. Progress in exosome engineering, encompassing surface changes and augmented targeting abilities, has significantly expanded their therapeutic potential. Nonetheless, exosome‐based therapeutics are still in the preliminary phases of clinical investigation, with only three clinical trials presently underway (two for AD and one for ALS).

Our research group is also conducting research on the therapeutic effects of exosomes derived from stem cells, antioxidant‐loaded enriched exosomes, as well as transgenic exosomes expressing proteins potentially effective against neurodegeneration, in both in vitro and in vivo models of AD and PD.

Future initiatives should focus on broadening clinical trials to encompass more NDs, such as PD and HD, while also addressing the issues of scalability and standardization in exosome production. The rising interest in exosome‐based therapeutics, alongside technological progress and an enhanced comprehension of their therapeutic potential, is anticipated to propel revolutionary treatment techniques for NDs. These advancements may facilitate more accurate, efficient, and less invasive procedures, thereby enhancing patient outcomes in these NDs.

## Author Contributions

S.I., S.O., B.Y.K., S.R.M.S., and N.M.E.S. wrote the manuscript; S.I. designed the research; S.I., S.O., B.Y.K., S.R.M.S., and N.M.E.S. performed the research; S.I. and B.Y.K. analyzed the data.

## Disclosure

No funding was received for this work.

## Conflicts of Interest

The authors declare no conflicts of interest.

## Data Availability

The data that support the findings of this study are available from the corresponding author upon reasonable request.
